# Decreased locus coeruleus signal associated with Alzheimer’s disease based on neuromelanin-sensitive magnetic resonance imaging technique

**DOI:** 10.3389/fnins.2022.1014485

**Published:** 2022-10-05

**Authors:** Meng Li, Shanwen Liu, Hongqin Zhu, Zhiwen Guo, Yuqi Zhi, Rong Liu, Zhen Jiang, Xiaoyun Liang, Hua Hu, Jiangtao Zhu

**Affiliations:** ^1^Department of Radiology, The Second Affiliated Hospital of Soochow University, Suzhou, Jiangsu, China; ^2^Department of Neurology, The Second Affiliated Hospital of Soochow University, Suzhou, Jiangsu, China; ^3^Center for Molecular Imaging and Nuclear Medicine, State Key Laboratory of Radiation Medicine and Protection, School for Radiological and Interdisciplinary Sciences (RAD-X), Collaborative Innovation Center of Radiation Medicine of Jiangsu Higher Education Institutions, Soochow University, Suzhou, China; ^4^Institute of Artificial Intelligence and Clinical Innovation, Neusoft Medical Systems Co., Ltd., Shanghai, China; ^5^Florey Institute of Neuroscience and Mental Health, The University of Melbourne, Melbourne, VIC, Australia

**Keywords:** Alzheimer’s disease, locus coeruleus, neuromelanin, MRI, cognition

## Abstract

**Objective:**

Neuromelanin-sensitive magnetic resonance imaging (NM-MRI) technique was used to detect the changes of the locus coeruleus (LC) signals in Alzheimer’s disease patients (AD), and to analyze its correlation with cognitive function.

**Materials and methods:**

A total of 27 patients with AD, 15 patients with mild cognitive impairment (MCI), and 25 healthy controls (HC) were examined by NM-MRI technique. ImageJ software was used to measure the LC signals. The locus coeruleus signal contrast ratios (LC-CRs) were calculated, along with the measurement of neuropsychological scales.

**Results:**

The LC-CRs of AD patients were significantly different from that of HC (*p* = 0.007, 95% CI: −0.053∼−0.007). However, such significant differences were not observed between MCI and HC (*p* = 1.000, 95% CI: −0.030∼0.024), AD and MCI (*p* = 0.050, 95% CI: −0.054∼0.000). Furthermore, a significant positive correlation was identified between LC-CRs and MMSE sub item Drawing (*r* = 0.484, *p* = 0.011) in the AD group, MoCA sub item Attention (*r* = 0.519, *p* = 0.047) in the MCI group. The area under the curve of LC-CRs in the diagnosis of AD was 0.749 (*p* = 0.002, 95% CI: 0.618∼0.880), with a sensitivity of 85.2% and a specificity of 56.0%.

**Conclusion:**

The NM-MRI technique could quantify the pathological degenerations of the LC in AD. Such LC degenerations can be employed to distinguish AD from healthy elderly.

## Introduction

The locus coeruleus (LC) is a short rod-shaped bilaterally symmetrical neural nucleus located in the pontine tegmentum (PT), which is at the lateral border of the bottom of the fourth ventricle. Importantly, the LC is the main site for the synthesis of norepinephrine in the brain (Aston-Jones and Cohen, 2005). Previous studies suggest that LC degeneration plays a key role during the development of Alzheimer’s disease (AD), and LC may be the first area of neurofibrillary tangles (NFTs) and Tau protein deposition before cognitive impairment and clinical symptoms appear in AD patients (Braak et al., 2011; Theofilas et al., 2017). It is well accepted that timely intervention is key to delay the development of the disease, which emphasizes the importance of diagnosis of early AD. Biomarkers for AD diagnosis are various. Among them, body fluid markers such as cerebrospinal fluid amyloid-β (Aβ) and Tau protein are invasive and difficult to obtain. Imaging markers such as Positron Emission Computed Tomography (PET) have certain radiation hazards and are expensive, which may not be accessible for diagnosis of early AD.

Neuromelanin-sensitive magnetic resonance imaging (NM-MRI) is mostly used in the detection of substantia nigra and LC in Parkinson’s disease (Sasaki et al., 2006; Kashihara et al., 2011; Sulzer et al., 2018). The norepinephrine neurons of the LC contain neuromelanin (NM). NM mainly contains melanin, metal ions, and other substances, of which the metal ions are mainly iron. The melanin-iron complex has paramagnetic properties, which can shorten the T1 relaxation time (Enochs et al., 1997; Keren et al., 2015). The image after scanning with NM-MRI technique shows a relatively high signal, which can clearly show the LC. In recent years, the NM-MRI technique has been applied to the study of AD patients, and some studies have shown that the LC signal of AD patients is lower than that of healthy controls (HC) (Hou et al., 2021). However, this technique has been rarely employed to investigate the LC signal changes among HC, AD, and MCI and their correlation with cognitive function.

We put forward the following hypotheses: (1) The signal intensity of LC in AD patients was significantly lower than that of HC and or MCI. (2) The signal intensity of LC in MCI patients was significantly lower than that of HC. (3) There was a correlation between LC signal intensity and cognitive function. Therefore, in this study, the NM-MRI technique was used to measure the signal intensity of the LC, and to investigate the correlation between LC degeneration and cognitive function, aiming to find a convenient, affordable, and non-invasive imaging biomarker to provide a basis for the diagnosis of AD.

## Materials and methods

### Participants

The experiment protocol was reviewed and approved by the Ethics Committee of the Second Affiliated Hospital of Soochow University. Informed consent forms were obtained from all participants, including 27 AD patients and 15 MCI patients diagnosed in the memory disorder clinic of the Neurology Department of the Second Affiliated Hospital of Soochow University from November 2020 to December 2021, as well as 25 gender and age matched healthy volunteers that were recruited from local communities.

### Neuropsychometric measures

The researchers were trained to test specialized scales. The participants took neuropsychological tests in a quiet room. Cognitive function was tested using the Mini-Mental State Examination (MMSE) and the Montreal Cognitive Assessment (MoCA). MMSE scale includes 11 sub items: Time orientation, Place orientation, Instantaneous memory, Attention and numeracy, Delayed memory, Naming, Retelling, Executive function, Reading, Writing, and Drawing. MoCA scale includes seven sub items: Visuospatial and executive function, Naming, Attention, Language, Abstraction, Delayed memory, and Orientation.

**FIGURE 1:**
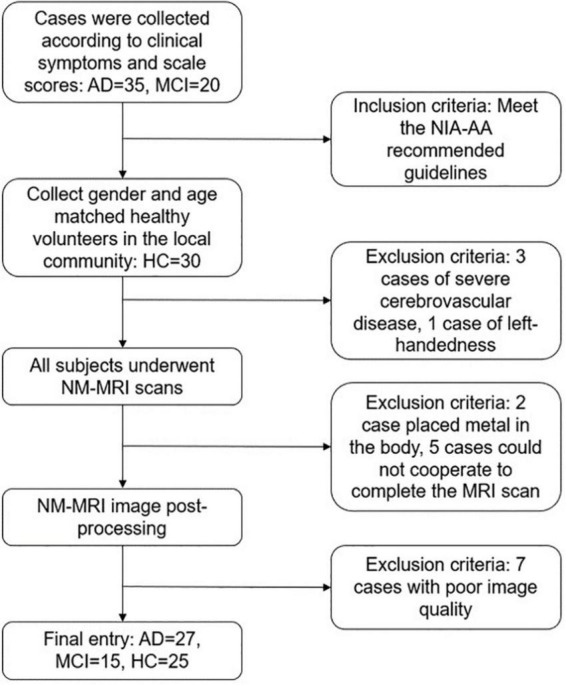
Inclusion and exclusion criteria flow diagram.

Inclusion and exclusion criteria flow of the study is shown in diagram (Figure 1). Inclusion criteria: (1) Meet the core diagnostic criteria for probable AD dementia jointly developed by the National Institute on Aging (NIA) and Alzheimer’s Association (AA) in 2011 (McKhann et al., 2011). (2) The diagnostic criteria for mild cognitive impairment (MCI) also refer to the NIA-AA recommended guidelines: subjective and objective cognitive impairment, normal activities of daily living, and not yet meeting the criteria for dementia (Albert et al., 2011). (3) Age 60∼85 years old, right-handed. (4) Able to read and understand the content of the research scale. Exclusion criteria: (1) Other diseases that cause cognitive impairment, such as cerebrovascular disease, brain tumor, Hachinski ischemic score ≥ 4 points. (2) Severe medical diseases, such as heart, lung, liver, and kidney insufficiency, hypothyroidism, malignant tumor, and other chronic wasting sexually transmitted diseases. (3) Severe depression, schizophrenia, and other mental illnesses. (4) Those who cannot cooperate with the scale evaluator. (5) Those who have contraindications for magnetic resonance examination.

### Magnetic resonance imaging acquisition

**FIGURE 2:**
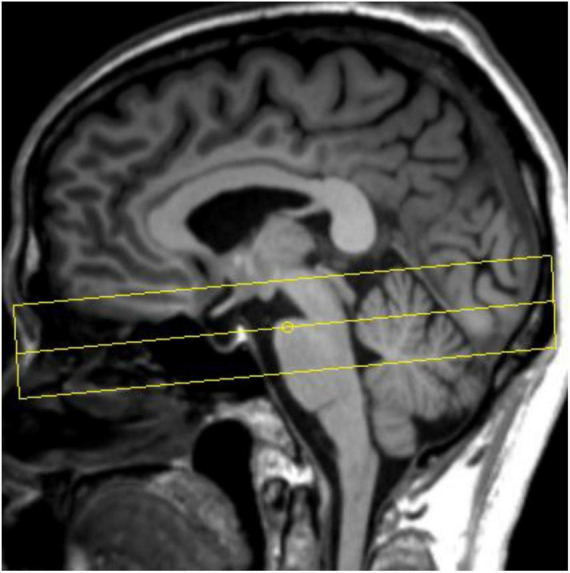
Schematic diagram of neuromelanin-sensitive magnetic resonance imaging (NM-MRI) scan.

All participants underwent cranial MRI scans on a 3T MR scanner (Prisma, Siemens Healthcare, Erlangen, Germany) with a 64-channel phased array head coil. Routine sequence scans were first performed, including T1WI, T2WI, and diffusion weighted imaging (DWI), to exclude participants with structural abnormalities of the brain. Sagittal 3D-T1WI volumetric data were subsequently acquired using T1-weighted three-dimensional magnetization-prepared rapid acquisition gradient-echo sequence (3D MP-RAGE). The schematic diagram of NM-MRI scan is shown in Figure 2. NM-MRI imaging scan is performed: TR = 262.0 ms, TE = 3.01 ms, flip angle = 40°, 8 signal averages, FOV = 200 mm× 200 mm, voxel size = 0.6 mm× 0.6 mm× 3.0 mm, 10 slices, acquisition time 11 min 11 s.

### The calculation of the locus coeruleus

**FIGURE 3:**
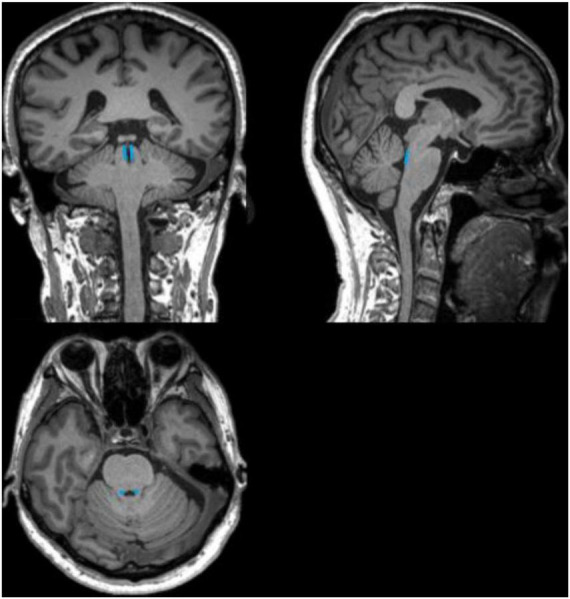
Schematic diagram of the anatomical location of the locus coeruleus (LC). The schematic diagram shows coronal, sagittal, and axial 3D-T1WI images from a 71-year-old healthy control, with the anatomical location of the LC outlined in blue.

**FIGURE 4:**
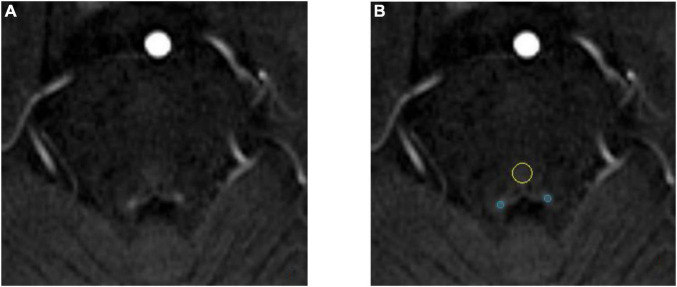
Schematic diagram of locus coeruleus (LC) signal and the region of interest (ROI) delineation. Both images are from the same 71-year-old healthy control. **(A)** The locus coeruleus (LC) exhibits relatively high signal after NM-MRI scanning. **(B)** Schematic diagram of the region of interest (ROI). The blue circle is the LC and the yellow circle is the pontine tegmentum (PT) reference area.

The anatomical location of the LC is shown in Figure 3. As shown in the Figure 4A, on the scanned axial map of the pons, the LC shows the anterior dorsum of the pons and two small hyperintense areas around the base of the fourth ventricle. By virtue of the anatomical position, the slice on which LC showed the maximum signal intensity was determined LC. By using the ImageJ software,^1^ a circular region of interest (ROI) with an area of 1 mm^2^ was drawn on the brightest pixels of the LC on the left and right sides, respectively, and the average signal intensities were calculated from the LCs on both sides. Using the signal intensity of the pontine tegmentum (SIPT) as a reference area, it was calculated using a circular ROI of 10 mm^2^ (Figure 4B). In this study, the division of the regions of interest and the calculation method of locus coeruleus signal contrast ratios (LC-CRs) were as follows: The circular ROI was fixed at each pons, that is, at approximately the same distance from the LC ROIs on both sides, which was performed based on previous studies. Signal intensities of the LC (SILCs) were measured on both sides, respectively, followed by the calculation of the average SILC. Finally, the LC signal contrast was calculated with the formula: LC-CRs = (SILC-SIPT)/SIPT.

### Statistical analysis

SPSS 26.0 software package was used for statistical analysis (IBM, Armonk, NY, USA). Gender variables were expressed as percentages, and comparisons between groups were performed using the χ^2^ test. The age and LC-CRs values for the three groups were expressed as mean ± standard deviation, and analyzed by analysis of variance (ANOVA). *Post-hoc* analysis was further performed to investigate whether there was any significant difference between any two groups of LC-CRs data with the Bonferroni correction method applied for correcting multiple comparisons. The disease duration, MMSE score, MoCA score, and SIPT data were expressed as the median [M (P25, P75)]. The Wilcoxon–Mann–Whitney test was used for the comparison of disease duration. The SIPT data, MMSE score and MoCA score, was compared using Kruskal–Wallis *H* test. Spearman correlation analysis was performed between LC-CRs and neuropsychological scale scores. Receiver operating characteristic (ROC) curve analysis was used to calculate the efficacy of LC-CRs values in diagnosing AD, which was measured by area under the ROC curve (AUC) value, sensitivity and specificity. *P* < 0.05 was considered statistically significant.

## Results

### Comparison of clinical data of participants

No significant differences in gender and age distribution were detected among the three groups (χ^2^ = 3.057, *p* = 0.217; *F* = 1.828, *p* = 0.169). There was no significant difference in disease duration between AD group and MCI group (*Z* = −1.406, *p* = 0.160). Significant differences were found among the three groups in the MMSE and MoCA total scores and their sub items, except for the MMSE sub item Naming and Drawing (*H* = 1.386, *p* = 0.500; *H* = 5.591, *p* = 0.061, respectively) (Table 1).

**TABLE 1:** Demographic characteristics, cognitive scores, and imaging measures of study subjects.

Characteristic	AD (*n* = 27)	MCI (*n* = 15)	HC (*n* = 25)	Statistics	*P*-value
**Gender (*n*, %)**					
Male	6 (22.2%)	4 (26.7%)	11 (44.0%)	χ^2^ = 3.057	0.217
Female	21 (77.8%)	11 (73.3%)	14 (56.0%)		
Age Mean ± SD	70.4 ± 9.2	70.4 ± 9.7	66.2 ± 7.2	*F* = 1.828	0.169
Disease duration (months) M (P_25,_P_75_)	36 (18, 48)	24 (9, 36)	NA	*Z* = –1.406	0.160
MMSE score M (P_25,_P_75_)	20 (14, 24)	27 (25, 28)	28 (27, 29)	*H* = 50.047	0.000**
Time orientation	3 (1, 4)	4 (3, 5)	5 (5, 5)	*H* = 35.248	0.000**
Place orientation	3 (3, 4)	5 (4, 5)	5 (5, 5)	*H* = 27.973	0.000**
Instantaneous memory	3 (2, 3)	3 (3, 3)	3 (3, 3)	*H* = 19.084	0.000**
Attention and numeracy	4 (1, 5)	5 (4, 5)	5 (4.5, 5)	*H* = 12.564	0.002**
Delayed memory	0 (0, 1)	2 (1, 3)	3 (2, 3)	*H* = 32.516	0.000**
Naming	2 (2, 2)	2 (2, 2)	2 (2, 2)	*H* = 1.386	0.500
Retelling	1 (0, 1)	1 (1, 1)	1 (1, 1)	*H* = 7.181	0.028*
Executive function	2 (2, 2)	3 (2, 3)	3 (3, 3)	*H* = 20.963	0.000**
Reading	1 (0, 1)	1 (1, 1)	1 (1, 1)	*H* = 11.407	0.003**
Writing	1 (0, 1)	1 (1, 1)	1 (1, 1)	*H* = 15.894	0.000**
Drawing	1 (0, 1)	1 (1, 1)	1 (1, 1)	*H* = 5.591	0.061
MoCA score M (P_25,_P_75_)	14 (9, 20)	22 (19, 24)	27 (26, 28)	*H* = 52.353	0.000**
Visuospatial and executive function	2 (1, 3)	4 (3, 5)	5 (4, 5)	*H* = 26.273	0.000**
Naming	2 (1, 3)	3 (2, 3)	3 (3, 3)	*H* = 19.386	0.000**
Attention	5 (2, 6)	6 (6, 6)	6 (5.5, 6)	*H* = 16.560	0.000**
Language	1 (0, 2)	2 (1, 2)	2 (2, 3)	*H* = 11.539	0.003**
Abstraction	0 (0, 1)	2 (1, 2)	2 (2, 2)	*H* = 24.252	0.000**
Delayed memory	0 (0, 0)	0 (0, 3)	4 (3, 5)	*H* = 45.412	0.000**
Orientation	3 (2, 5)	5 (5, 6)	6 (6, 6)	*H* = 28.468	0.000**
SIPT M (P_25,_P_75_)	90.76 (88.14, 94.39)	88.49 (86.48, 90.54)	86.86 (85.31, 90.69)	*H* = 5.950	0.051
LC-CRs Mean ± SD	0.1271 ± 0.0291	0.1539 ± 0.0433	0.1571 ± 0.0324	*F* = 5.853	0.005**

AD, Alzheimer’s disease; MCI, mild cognitive impairment; HC, health control; MMSE, Mini-Mental State Examination; MoCA, Montreal Cognitive Assessment; STPT, pontine tegmentum signal intensity; LC-CRs, locus coeruleus contrast ratios; NA, not applicable, *P < 0.05, **P < 0.01.

### Comparison of locus coeruleus signal contrast ratios among the three groups

Analysis of variance analysis revealed a statistically significant difference in LC-CRs among the three groups (*F* = 5.853, *p* = 0.005). To exclude the influence of the ROI signal intensity in the selected pontine tegmental reference area, the SIPT values were compared among the three groups. No significant difference of SIPT was observed among the three groups (*H* = 5.950, *p* = 0.051), indicating that the LC-CRs signal difference was independent on the SIPT (Table 1).

*Post-hoc* analysis showed that AD group had significantly lower LC-CRs than HC group (*p* = 0.007, 95% CI: −0.053∼−0.007). However, such a significant difference in LC-CRs was not observed between MCI group and HC group, AD group, and MCI group (*p* = 1.000, 95% CI: −0.030∼0.024; *p* = 0.050, 95% CI: −0.054∼0.000, respectively) (Table 2).

**TABLE 2:** *Post-hoc* multiple comparisons of locus coeruleus signal contrast ratios (LC-CRs) between groups.

Between-group comparison	95% confidence interval		*P*-value
	
	Lower limit	Upper limit	
AD vs. MCI	−0.054	0.000	0.050
AD vs. HC	−0.053	−0.007	0.007**
MCI vs. HC	−0.030	0.024	1.000

**P < 0.01.

### Correlation analysis between locus coeruleus signal contrast ratios and cognitive function

The Spearman correlation analysis showed: in the AD group, a significant positive correlation was identified between LC-CRs and MMSE sub item Drawing (*r* = 0.484, *p* = 0.011), no significant correlation was found between LC-CRs and MMSE, MoCA total score and other sub items (all *p* > 0.05). In the MCI group, LC-CRs were positively correlated with MoCA sub item Attention (*r* = 0.519, *p* = 0.047), similarly, there was no significant correlation between LC-CRs and MMSE, MoCA total score, and other sub items (all *p* > 0.05). In the HC group, no significant correlation was found between LC-CRs and MMSE, MoCA total score or sub items (all *p* > 0.05) (Tables 3, 4).

**TABLE 3:** Correlation analysis between locus coeruleus signal contrast ratios (LC-CRs) and Mini-Mental State Examination (MMSE) including its sub items.

MMSE and its sub items	AD		MCI		HC	
			
	*r*-value	*P*-value	*r*-value	*P*-value	*r*-value	*P*-value
MMSE	0.089	0.661	0.396	0.143	−0.005	0.980
Time orientation	0.080	0.692	0.283	0.306	−0.250	0.229
Place orientation	−0.065	0.749	−0.098	0.728	−0.106	0.614
Instantaneous memory	−0.045	0.822	NA	NA	NA	NA
Attention and numeracy	0.106	0.597	0.387	0.154	−0.037	0.860
Delayed memory	−0.334	0.088	0.170	0.545	0.076	0.718
Naming	0.277	0.162	NA	NA	0.041	0.846
Retelling	0.286	0.149	0.000	1.000	0.000	1.000
Executive function	0.002	0.992	−0.033	0.908	0.055	0.792
Reading	0.065	0.747	NA	NA	NA	NA
Writing	0.106	0.597	NA	NA	0.028	0.893
Drawing	0.484	0.011*	0.409	0.131	−0.076	0.719

*P < 0.05, NA, not applicable (One set of data is constant).

**TABLE 4:** Correlation analysis between locus coeruleus signal contrast ratios (LC-CRs) and Montreal Cognitive Assessment (MoCA) including its sub items.

MoCA and its sub items	AD		MCI		HC	
			
	*r*-value	*P*-value	*r*-value	*P*-value	*r*-value	*P*-value
MoCA	0.117	0.561	0.483	0.068	0.365	0.073
Visuospatial and executive function	0.291	0.141	0.111	0.693	0.014	0.949
Naming	−0.214	0.283	0.085	0.763	−0.136	0.516
Attention	0.086	0.671	0.519	0.047*	0.248	0.232
Language	0.124	0.537	0.345	0.207	0.028	0.893
Abstraction	0.103	0.610	0.144	0.608	0.069	0.742
Delayed memory	0.000	1.000	0.150	0.595	0.389	0.055
Orientation	0.028	0.892	0.171	0.542	−0.250	0.229

*P < 0.05.

### Receiver operating characteristic curve analysis of the efficacy of locus coeruleus signal contrast ratios in the diagnosis of Alzheimer’s disease

**FIGURE 5:**
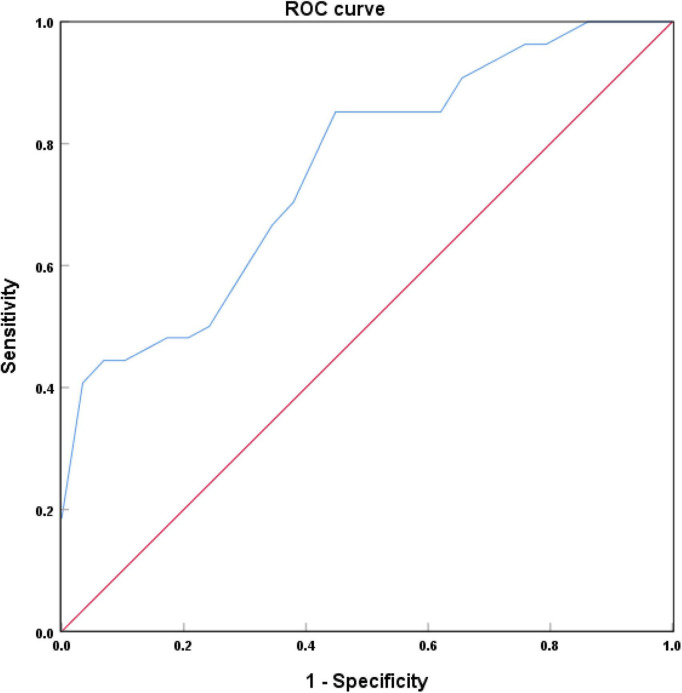
Receiver operating characteristic (ROC) curve of locus coeruleus (LC) signal contrast ratios in Alzheimer’s disease (AD) and healthy controls (HC).

Figure 5 showed that the area under the curve (AUC) of LC-CRs in the diagnosis of AD was 0.749 (*P* = 0.002, 95% CI: 0.618∼0.880). When the maximum value of the Youden index was 0.412, the corresponding LC-CRs value was 0.1531, which can be used as the critical value of LC-CRs to diagnose AD, the diagnostic sensitivity is 85.2%, and the specificity is 56.0%.

## Discussion

In this study, NM-MRI technique was used to quantitatively measure the signal intensity of LC. The results showed that the LC signal intensity of AD patients was significantly lower than that of HC. As mentioned in the introduction section above, the images showed relatively high signal in the LC because of the presence of neuromelanin. Thus, the significant attenuation of the LC signal implied that there were reductions of neuromelanin as well as neuronal degeneration in LC among AD patients. No statistically significant differences were detected in LC signal intensity whether compared between AD and MCI or between HC and MCI, which may be due to the relatively small sample size or the absence of significant LC degenerations *per se*. This question will be further explored in future studies with a larger sample size.

Contrary to our hypothesis, no significant correlation was found between LC-CRs and MMSE, MoCA total scores, which was consistent with Takahashi et al. (2015). However, Hou et al. (2021) found that LC signal was correlated with MMSE score, which may be caused by differences in ROI delineation, scanning sequence parameters, and sample size. Further analyzes of the sub items of MMSE and MoCA revealed that LC-CRs were significantly positively correlated with Drawing (in AD group) and Attention (in MCI group). Specifically, drawing reflects visuospatial ability, i.e., impairment of visuospatial ability is significantly positively correlated with LC degeneration, which was in line with the findings of Kelly et al. (2017). LC-norepinephrine system is involved in the regulation of attention, memory, and learning ability (Robbins, 1984; Benarroch, 2009). Based on the current research, it can be said that there is a certain correlation between LC and visuospatial ability and attention, but these abilities are only some aspects of cognitive function, therefor the relationship between LC and cognitive function needs to be further studied in the future.

Extracellular deposition of amyloid-β (Aβ) and NFTs caused by hyperphosphorylation of tau protein are two pathological features of AD (Braak and Braak, 1991; Weksler et al., 2005). A recent study showed a significant correlation between LC signal attenuation and cerebrospinal fluid Aβ levels in AD patients (Betts et al., 2019). Deposition of hyperphosphorylated tau protein in LC has been observed in MCI patients and the early stages of AD (Grudzien et al., 2007). Mounting evidence demonstrated that LC is an important part of the synthesis of norepinephrine in the brain (Berridge and Waterhouse, 2003; Samuels and Szabadi, 2008; Ross et al., 2015). LC degenerations indicated that the synthesis and secretion of norepinephrine in the brain were reduced. Along with the fact that norepinephrine is considered to be closely related to cognitive function in AD patients (Berridge and Waterhouse, 2003; Holland et al., 2021), it suggests that cognitive decline may be manifested as LC degenerations. If the LC signal decline can be detected during the early stage of AD, such cognitive decline may be alleviated or even avoided if norepinephrine-related treatment is applied promptly (Friedman et al., 1999; Holland et al., 2021; Matchett et al., 2021).

In this study, the ROC curve was used to analyze the efficacy of LC-CRs in diagnosing AD. The results showed that the AUC of LC-CRs in discriminating between AD and HC was 0.749, which was comparable to the results of Takahashi et al. (2015). The AUC value of the diagnostic model constructed by Dordevic et al. (2017) using NM-MRI technique was 0.86, the sensitivity was 100%, and the specificity was 80%, which was higher than those that of the present study. The disparity is likely due to the fact that they included the signal intensity on the left and right sides of each axial slice of the LC into the analysis, and did not take the average signal intensity of the two sides for analysis. The AUC value can be interpreted as the probability that AD patients are correctly diagnosed, and the results of the present study showed that LC-CRs have good diagnostic performance. This suggests that LC signal decline may be helpful to assist clinicians in the diagnosis of AD.

There is no consensus as to whether there is physiological degeneration of LC in normal aging population. During the earlier time, it was believed that the LC signal intensity gradually increased with age, reaching a peak around the age of 60, and then gradually decreased significantly (Manaye et al., 1995; Shibata et al., 2006). In recent years, some studies found that there was no significant difference in LC signal intensity among normal aging populations (Galgani et al., 2021; Giorgi et al., 2021). Presumably, it is because the former (Manaye et al., 1995; Shibata et al., 2006) had a large age span, involving the young, middle-aged, and the elderly, whereas the latter (Galgani et al., 2021; Giorgi et al., 2021) only studied the relationship between LC signal and age in the elderly aged 60–80. A stereological study of the human postmortem brain showed that normal aging does not affect LC volume and neuronal population, excluding physiological degeneration of LC (Theofilas et al., 2017). In this study, to avoid the effect of age on LC signal, the ages among the three groups were matched (i.e., no significant age difference among the three groups). During screening in the elderly at high risk for Alzheimer’s disease, the decrease of LC signal intensity suggested that there were pathological changes in LC given that the normal physiological degeneration may be excluded according to the literature reports in recent years. At this time, early intervention can slow down the progress of the disease to a certain extent.

This study has certain limitations. Firstly, the classification of AD, MCI, and healthy control groups is based on clinical psychological scales and clinical symptoms, and there is no pathological basis, such as cerebrospinal fluid biomarkers or PET. However, through assessments, we excluded dementia caused by other neurodegenerative diseases as much as possible. Secondly, the strongest level of the LC signal is based on the observation, and the average value of the LC signal strength on the left and right sides is calculated, which may have some errors. Thirdly, the final sample size of this study was relatively small and the results are from single center rather than from multiple centers. Future studies are warranted to address these issues.

## Conclusion

In the present study, it was demonstrated that NM-MRI technique could quantify the pathological degenerations of the LC in the AD cohort. The significantly reduced LC signal in the AD cohort compared with HC suggests that LC degenerations can be employed to distinguish AD from healthy elderly. In the future, it is necessary to conduct further research on whether the decrease of LC signal can be used as a biomarker for AD diagnosis.

## Data availability statement

The raw data supporting the conclusions of this article will be made available by the authors, without undue reservation.

## Ethics statement

The studies involving human participants were reviewed and approved by Ethics Committee of the Second Affiliated Hospital of Soochow University. The patients/participants provided their written informed consent to participate in this study.

## Author contributions

ML designed the study, analyzed the data, and drafted the manuscript. SL collected and organized data. HZ processed the image. ZG and YZ collected data. XL and JZ edited and reviewed the manuscript. RL and ZJ provided funding supports. HH and JZ contributed to the conception and design of the study. All authors contributed to the article and approved the submitted version.

## Footnotes

1
https://imagej.nih.gov/ij

## Abbreviations

LC: locus coeruleus
PT: pontine tegmentum
AD: Alzheimer’s disease
NM-MRI: neuromelanin-sensitive magnetic resonance imaging
MCI: mild cognitive impairment
HC: healthy controls
MMSE: Mini-Mental State Examination
MoCA: Montreal Cognitive Assessment
DWI: diffusion weighted imaging
ROI: region of interest
SIPT: signal intensity of the pontine tegmentum
LC-CRs: locus coeruleus signal contrast ratios
SILC: signal intensity of the locus coeruleus
ANOVA: analysis of variance
ROC: receiver operating characteristic
AUC: area under the curve.
